# The association of the TLR3 SNP rs3775291 with schizophrenia is influenced by age

**DOI:** 10.3389/fgene.2026.1839603

**Published:** 2026-05-20

**Authors:** Yulong Wang, Lin Peng, Yue Liu, Ziang Gao, Jinghan Wang, Tianxiang Zhang, Yanlong Wang

**Affiliations:** 1 College of Teacher Education, Qilu Normal University, Jinan, Shandong, China; 2 Research Institute of Mental Health, Jining Medical University, Jining, Shandong, China; 3 Lin He’s Academician Workstation of New Medicine and Clinical Translation, Jining Medical University, Jining, Shandong, China

**Keywords:** age, Rs3775291, schizophrenia, SNP, TLR

## Abstract

**Background:**

Toll-like receptor 3 (TLR3) plays an important role in the pathogenesis of schizophrenia and the *TLR3* single nucleotide polymorphism (SNP) rs3775291 is associated with central nervous system diseases. In this study, we analysed the association between rs3775291 and schizophrenia.

**Methods:**

A case-control study was performed that included 394 patients with schizophrenia and 445 healthy controls. Peripheral venous blood was collected, whole-genome DNA was extracted, and rs3775291 polymorphisms were detected using PCR and direct sequencing. Differences in distribution of allele and genotype frequencies between the two groups were compared using co-dominant, dominant, and recessive genetic models. The effects of sex, age, and family history were also analysed.

**Results:**

Allele and genotype frequencies of rs3775291 were not associated with schizophrenia overall, nor were they associated with schizophrenia in the different sex or family history groups (P > 0.05). However, in the < 40 years age group, co-dominant and dominant model analyses showed that the frequency of TC and CC genotypes in patients with schizophrenia was significantly higher than that in the control group (P < 0.05), and TC and CC genotypes were associated with susceptibility to schizophrenia. There was no significant difference among people older than 40 years.

**Conclusion:**

The effect of rs3775291 on schizophrenia was age-related, TC and CC genotypes were associated with susceptibility to schizophrenia in younger individuals, and the TT genotype had a protective effect regarding schizophrenia.

## Introduction

The main symptoms of schizophrenia include low mood, loss of pleasure, loss of will or motivation, and social withdrawal (Dong et al., 2025). The aetiology of schizophrenia is complex and involves many factors, including heredity and the environment. Increasing evidence suggests that changes in immune function are also associated with schizophrenia pathophysiology (Ermakov et al., 2025). It has been shown that immune activation and/or immunoinflammatory responses during neurodevelopment can contribute to the onset and progression of schizophrenia. Xu et al. (2025) found that prenatal exposure to polyinosinic-polycytidylic acid (Poly I:C)-induced maternal immune activation (MIA) in mice leads to schizophrenia-like abnormal behaviors in the offspring. Rat offspring exposed to Poly I:C exhibit a range of behavioral abnormalities associated with positive, negative, and cognitive symptoms of schizophrenia, including visuotactile memory deficits, object recognition impairment, and reduced social preference (Lins et al., 2019). Mechanistically, MIA activates the Toll-like receptor (TLR) pathway, leading to upregulation of cytokine expression in the offspring brain while also affecting oxidative stress and apoptotic pathways, ultimately resulting in long-term alterations in synaptic plasticity (Su et al., 2022).

TLRs, a class of natural receptors, are pattern-recognition molecules that induce inflammation and are connected to the innate and adaptive immune systems through adaptor proteins and downstream elements. TLR central nervous system involvement includes both normal function and pathogenesis (Saleki et al., 2024). McKernan et al. (2011) cultured the peripheral blood of 40 patients with schizophrenia, 20 with bipolar disorder, and 40 healthy controls in the presence of TLR agonists for 24 h to determine the activity of TLR agonist-induced cytokines. They found that specific TLR ligand-mediated abnormalities in cytokine release are associated with immune dysfunction in patients with schizophrenia. Furthermore, disruption of TLR/mTOR signalling leads to insufficient neurodevelopment and synaptic plasticity, which are the major symptoms of schizophrenia (Lashgari et al., 2024).

The TLRs comprise 13 families. Changes in the expression of TLRs in peripheral blood mononuclear cells of patients with schizophrenia has been analysed using quantitative reverse transcription polymerase chain reaction (PCR). TLR1, TLR2, TLR4, TLR6, and TLR9 expression levels decrease in patients with schizophrenia, while the expression levels of TLR3 and TLR7 increase (Kozłowska et al., 2019). These changes in gene expression can be regulated by treatment with antipsychotics (Kéri et al., 2017). Müller et al. (2012) studied the differences in the expression of toll-like receptors TLR2, TLR3, and TLR4 on CD14^+^ monocytes of 31 patients with schizophrenia and 31 healthy controls, using lipopolysaccharide (LPS) stimulation to simulate bacterial infection and Polycan/Poly I:C stimulation to simulate viral infection. The concentration of interleukin-1β (IL-1β) in CD33^+^ monocytes before and after stimulation was also detected. While the expression levels of TLR3 and TLR4 are significantly increased in patients with schizophrenia compared to healthy controls, the expression levels of TLR2 are not significantly different; however, the expression levels of both TLR3 and TLR4 decrease after LPS or Polycan/Poly I:C stimulation. The concentration of IL-1β in the CD33^+^ monocytes of patients with schizophrenia was significantly lower before and after Poly I:C stimulation compared to that in healthy controls, and the concentrations decreased after LPS stimulation (Müller et al., 2012). This suggests that higher TLR3 and TLR4 expression may compensate for functional deficits in patients with schizophrenia, while the lower concentration of IL-1β may reflect diminished monocyte function in the patients.

TLR3 can play an important role in the pathogenesis of schizophrenia. Chen et al. (2017) found that TLR3 downregulates *Disc1* expression in patients with schizophrenia through MYD88 to control neuronal morphology. In the Poly I:C-mediated maternal immune activation (MIA) model, TLR3 serves as a key sensor of pathogen-associated molecular patterns (PAMPs) for the innate immune system. Upon stimulation, TLR3 drives downstream inflammatory pathways, leading to increased expression of pro-inflammatory cytokines and promoting glial cell maturation and differentiation. This pathway represents an important environmental risk mechanism for schizophrenia (Bergdolt and Dunaevsky, 2019). It has been reported that Poly I:C binding to TLR3 activates pro-inflammatory signaling and elevates the levels of pro-inflammatory cytokines, which can cross the placental barrier and the blood-brain barrier, thereby inducing peripheral inflammation and neuroinflammation in the offspring (Su et al., 2022).

The single nucleotide polymorphism (SNP) rs3775291 is located in the fourth exon of TLR3 gene, and its variant is a missense mutation (C1234T in mRNA, L412F in protein) that reduces the binding capacity of TLR3 to double-stranded RNA by 49% (Zhou et al., 2011). Individuals carrying the T allele (mutant type) exhibit significantly elevated serum interferon-alpha (IFN-α) levels (Ferreira et al., 2025) and reduced overall activation capacity of the TLR3-NF-κB pathway (Zhou et al., 2011). It has been found that rs3775291 is associated with tick-borne encephalitis virus (TBEV), hepatitis C virus (HCV), and hepatitis B virus (HBV) infections, as well as other diseases. Barkhash et al. studied the association between tick-borne encephalitis susceptibility and SNP rs3775291 in a Russian population and found that the frequency of C alleles and C/C homozygotes at this site was significantly higher in the patients than in the controls (Barkhash et al., 2013). This suggests that the C allele of TLR3 rs3775291 is associated with susceptibility to TBEV-induced central nervous system disease. Meanwhile, Geng et al. found that HCV infection is associated with the rs3775291 TT/CT genotype, and that the risk of infection is reduced for individuals with the T allele (Geng et al., 2016). A meta-analysis showed that TLR3 rs3775291 is associated with the risk of HBV infection, and allele T and genotype TT may increase susceptibility to HBV infection (Ye et al., 2020). Venkatasubramanian et al. proposed the “TRIPS hypothesis” of schizophrenia risk, which suggests that maternal prenatal infection may lead to progressive neurological changes in patients with schizophrenia in a way that is dependent on TLRs, especially TLR3 and TLR4 (especially TLR3/TLR4) (Venkatasubramanian and Debnath, 2013). Therefore, is there an association between TLR3 SNP rs3775291 and the risk of developing schizophrenia? Aflouk et al. included 260 schizophrenia patients, 260 controls, and 130 bipolar disorder patients (of Caucasian population) to examine the genetic susceptibility of rs3775291 in the two disorders and reported no significant risk association with schizophrenia (Aflouk et al., 2025). In the current study, we investigated the association between the TLR3 SNP rs3775291 and susceptibility to schizophrenia in a Chinese population.

## Materials and methods

### Study population

We recruited 394 patients with schizophrenia and 445 healthy controls. Clinical and demographic data were collected from the study participants.

All patients were unrelated individuals of Chinese descent recruited from the Shandong Daizhuang Hospital and the Genetic Resources Center of Jining Medical University. The diagnosis of schizophrenia was established based on the presence of relevant symptoms (ICD-10 code: F20.0). Patient recruitment followed strict inclusion and exclusion criteria. Inclusion criteria included the recruitment of volunteers diagnosed with schizophrenia who had been hospitalized at least once. The diagnosis was made by experienced psychiatrists according to the Diagnostic and Statistical Manual of Mental Disorders, Fourth Edition (DSM-IV); positive, negative, and general symptoms were assessed using the Positive and Negative Syndrome Scale (PANSS). Exclusion criteria included the exclusion of patients with hematological disorders, as well as those with other psychiatric disorders or general medical conditions. All patients were treated with typical and/or atypical antipsychotic medications, including first-generation (fluphenazine, haloperidol, chlorpromazine) or second-generation (risperidone, olanzapine, clozapine) agents, or a combination of both.

Healthy controls were recruited from the Affiliated Hospital of Jining Medical University. Inclusion criteria comprised the recruitment of healthy volunteers without any personal or family history of psychiatric disorders. However, subjects with hematological disorders or a diagnosis of inflammatory diseases were excluded.

This study was approved by the Medical Ethics Committee of Jining Medical University (JNMC-2023-YX-038), conducted in accordance with the Declaration of Helsinki of the World Medical Association, and all participants provided informed consent to participate.

### Collection of blood samples and genomic DNA extraction

A total of 5 mL of EDTA-anticoagulated venous blood was collected from each patient and healthy control subject. Genomic DNA was isolated from peripheral blood leukocytes using a TIANamp Blood DNA kit (Tiangen Biotech, Beijing, China) and stored at −80 °C at the Jining Medical University Genetic Resource Centre until further use. The concentrations and purities were determined for all extracted DNA samples. Only highly pure DNA samples with A260/A280 ratios within a 1.8–2.0 range and with concentrations ≥ 10 ng/μL were used in the study.

### PCR amplification, sequencing, and genotyping

Amplification of the *LTR3* region (NC_000004.12, 800bp) in the DNA samples was performed by PCR using TaKaRa Taq™ (Code No. R001A). Total PCR reaction volumes were 25 μL and included 200 ng DNA template, 2 μL dNTP Mixture, 0.5 μM each of upstream and downstream primers, and 0.13 μL Taq DNA polymerase. Primer sequence: forward 5′-TGT​CCA​CCA​CCA​GCA​ATA​CA-3′, reverse 5′-AGC​ATC​AGT​CGT​TGA​AGG​CT-3’. PCR cycling involved a single cycle of 94 °C for 5 min followed by 35 cycles of 94 °C for 30 s, 58 °C for 40 s, and 72 °C for 60 s with a final extension cycle of 72 °C for 7 min. The PCR assays were performed using a PCR Detection System (Eppendorf, Hamburg, Germany). For analysis, 5 μL of each PCR reaction was evaluated using 1.5% agarose gel electrophoresis with a λ-EcoT14 I/Bgl II digest (Code No. 3408) being used as the electrophoresis molecular weight standard. After electrophoresis, a Cytiva Chemiluminescence AI 800 gel imager (Cytiva, Marlborough, MA, United States) was used to visualize the amplification results. The PCR products with the correct predicted fragment size were directly sequenced using an ABI 3730xl DNA Sequencer (Applied Biosystems, Carlsbad, CA, United States) with BigDye™ Terminator v.3 kit (Applied Biosystems, Carlsbad, CA, United States) and the rs3775291 base composition of the samples analysed.

### Statistical analysis

G*power software (version 3.1.9.7) was used to estimate the total required sample size for the present study according to the following parameters: α error = 0.05, power = 0.80, and small effect size (Faul et al., 2007). Thus, a total of 788 subjects are required for this study.

The genotype distribution of rs3775291 in schizophrenia and control subjects were tested for correspondence (p > 0.05) to Hardy-Weinberg equilibrium (HWE) by Pearson’s χ^2^ test. The cross tabulation between SNP data and morphology was done using the statistical package for social science (IBM-SPSS) version 20 (Chicago, IL, United States). Analysis assuming co-dominant, dominant and recessive models was performed. The odds ratio and 95% Confidence Interval (CI) were calculated. The p-value was considered statistically significant if less than 0.05, and corresponding p-value while controlling for age, gender, and family history as co-variables.

## Results

### Clinical characteristics

The clinical and demographic data of the study participants are summarized in Table 1. There were 394 participants with schizophrenia (50% male, 50% female) and 445 healthy controls (56.2% male, 43.8% female). There was no significant difference in sex distribution between the two groups (P > 0.05). Participants with schizophrenia ranged in age from 14 to 81 years with a mean age of 48 years; 216 were younger than 40 years, and 178 were older than 40 years. The healthy control group ranged in age from 11 to 85 years with an average age of 38 years; 156 were younger than 40 years and 289 people were older than 40 years. The number of patients younger than 40 years in the schizophrenia group was higher than that in the control group, and the number of patients older than 40 years was lower than that in the control group. A total of 268 participants with schizophrenia provided information regarding a family history of schizophrenia of which 48 had a family history and 220 had no family history.

**TABLE 1:** Clinical and demographic data of participants with schizophrenia and healthy controls.

Project	SZ	HC	χ2	*p* value
Total number (n)	394	445	​	​
Male/female (n)	197/197	250/195	3.21	0.07
Age	14–81	11–85	​	​
Age ≤ 40/Age > 40 (n)	216/178	156/289	33.08	0.00
With family history/without family history (n)	48/220	​	​	​

HC, healthy control; SZ, participants with schizophrenia.

### Genetic association between rs3775291 and schizophrenia

Genotype distribution of rs3775291 in both schizophrenia (χ^2^ = 4.43, P = 0.11) and control subjects (χ^2^ = 0.27, P = 0.88) were consistent with the Hardy-Weinberg equilibrium (P > 0.05). The genotype and allele frequencies of rs3775291 in the patients with schizophrenia and healthy controls are shown in Table 2. For analyses of genotype frequency, co-dominant, dominant, and recessive genetic models were used. The results revealed no significant differences between the participants with schizophrenia and the healthy control groups (P > 0.05). The distribution of allele frequencies also showed no significant differences between patients with schizophrenia and controls [P > 0.05,OR = 1.06 (0.86–1.30)].

**TABLE 2:** Genotype and allele frequencies of rs3775291 in participants with schizophrenia and healthy controls.

Model	Genotype/allele	HC	SZ	χ^2^	*p* value	OR (95% CI)
Co-dominant	TT	51 (11.46%)	32 (8.12%)	3.43	0.18	​
	TC	170 (38.20%)	168 (42.64%)			
	CC	224 (50.34%)	194 (49.24%)			
Dominant	TT	51 (11.46%)	32 (8.12%)	2.61	0.11	​
	TC/CC	394 (88.54%)	362 (91.88%)			
Recessive	TT/TC	221 (49.66%)	200 (50.76%)	0.10	0.75	​
	CC	224 (50.34%)	194 (49.24%)			
Allele	T	272 (30.56%)	232 (29.44%)	0.25	0.62	1.06 (0.86–1.30)
	C	618 (69.44%)	556 (70.56%)			

HC, healthy control; SZ, participants with schizophrenia.

### Genetic association of rs3775291 with the clinical characteristics of schizophrenia

The effects of sex, age, and family history on the rs3775291 genotype and allele frequency distribution in patients with schizophrenia is shown in Tables 3–6. There was no significant difference in the genotype or allele frequency distribution of the rs3775291 locus between patients with schizophrenia and healthy controls (P > 0.05) based on co-dominant, dominant, and recessive model analyses in both male and female populations.

**TABLE 3:** Genotype frequency distribution (co-dominant model) of rs3775291 between patients with schizophrenia and healthy controls based on clinical characteristics.

Group	Genotype	HC	SZ	χ^2^	*p* value
Male	TT	29 (11.60%)	15 (7.61%)	3.26	0.2
	TC	92 (36.80%)	86 (43.65%)		
	CC	129 (51.60%)	96 (48.73%)		
Female	TT	22 (11.28%)	17 (8.63%)	0.78	0.68
	TC	78 (40.00%)	82 (41.62%)		
	CC	95 (48.72%)	98 (49.75%)		
Age ≤ 40	TT	26 (16.67%)	15 (6.94%)	8.87	0.01
	TC	60 (38.46%)	97 (44.91%)		
	CC	70 (44.87%)	104 (48.15%)		
Age > 40	TT	25 (8.65%)	17 (9.55%)	0.35	0.84
	TC	110 (38.06%)	71 (39.89%)		
	CC	154 (53.29%)	90 (50.56%)		
With family history	TT	51 (11.46%)	1 (2.08%)	4.08	0.13
	TC	170 (38.20%)	21 (43.75%)		
	CC	224 (50.34%)	26 (54.17%)		
Without family history	TT	51 (11.46%)	19 (8.64%)	2.41	0.3
	TC	170 (38.20%)	96 (43.64%)		
	CC	224 (50.34%)	105 (47.73%)		

HC, healthy control; SZ, participants with schizophrenia.

In individuals younger than 40 years of age, the co-dominant model revealed a significant difference in the rs3775291 genotype frequency between patients with schizophrenia and healthy controls (P < 0.05), and the TC and CC genotype frequencies were significantly higher in patients with schizophrenia than in healthy controls (Table 3). Consistent with the co-dominant model, the dominant model showed a significant difference in rs3775291 genotype frequency between the patients with schizophrenia and healthy control groups (P < 0.05), and the TC/CC genotype frequency was significantly higher in the schizophrenia group than that in the control group (Table 4). However, analyses of genotype (Table 5) and allele (Table 6) frequencies using the recessive model revealed no significant differences between patients with schizophrenia and healthy controls (P > 0.05). In individuals older than 40 years, there were no significant differences in genotype and allele distribution between patients with schizophrenia and healthy controls (P > 0.05).

**TABLE 4:** Genotype frequency distribution (dominant model) of rs3775291 between patients with schizophrenia and healthy controls based on clinical characteristics.

Group	Genotype	HC	SZ	χ^2^	*p* value
Male	TT	29 (11.60%)	15 (7.61%)	1.97	0.16
	TC/CC	221 (88.40%)	182 (92.39%)		
Female	TT	22 (11.28%)	17 (8.63%)	0.77	0.38
	TC/CC	173 (88.72%)	180 (91.37%)		
Age ≤ 40	TT	26 (16.67%)	15 (6.94%)	8.73	0.00
	TC/CC	130 (83.33%)	201 (93.06%)		
Age > 40	TT	25 (8.65%)	17 (9.55%)	0.11	0.74
	TC/CC	264 (91.35%)	161 (90.45%)		
With family history	TT	51 (11.46%)	1 (2.08%)	4.04	0.04
	TC/CC	394 (88.54%)	47 (97.92%)		
Without family history	TT	51 (11.46%)	19 (8.64%)	1.25	0.26
	TC/CC	394 (88.54%)	201 (91.36%)		

HC, healthy control; SZ, participants with schizophrenia.

**TABLE 5:** Genotype frequency distribution (recessive model) of rs3775291 between patients with schizophrenia and healthy controls based on clinical characteristics.

Group	Genotype	HC	SZ	χ^2^	*p* value
Male	TT/TC	121 (48.40%)	101 (51.27%)	0.36	0.55
	CC	129 (51.6%)	96 (48.73%)		
Female	TT/TC	100 (51.28%)	99 (50.25%)	0.04	0.84
	CC	95 (48.72%)	98 (49.75%)		
Age ≤ 40	TT/TC	86 (55.13%)	112 (51.85%)	0.39	0.53
	CC	70 (44.87%)	104 (48.15%)		
Age > 40	TT/TC	135 (46.71%)	88 (49.44%)	0.33	0.57
	CC	154 (53.29%)	90 (50.56%)		
With family history	TT/TC	221 (49.66%)	22 (45.83%)	0.25	0.61
	CC	224 (50.34%)	26 (54.17%)		
Without family history	TT/TC	221 (49.66%)	115 (52.27%)	0.4	0.53
	CC	224 (50.34%)	105 (47.73%)		

HC, healthy control; SZ, participants with schizophrenia.

**TABLE 6:** Allele frequency distribution of rs3775291 between patients with schizophrenia and healthy controls based on clinical characteristics.

Group	Allele	HC	SZ	χ^2^	*p* value	OR (95% CI)
Male	T	150 (30.00%)	116 (29.44%)	0.03	0.86	1.03 (0.77–1.37)
	C	350 (70.00%)	278 (70.56%)			
Female	T	122 (31.28%)	116 (29.44%)	0.31	0.58	1.09 (0.81–1.48)
	C	268 (68.72%)	278 (70.56%)			
Age ≤ 40	T	112 (35.90%)	127 (29.40%)	3.51	0.06	1.35 (0.99–1.83)
	C	200 (64.10%)	305 (70.60%)			
Age > 40	T	160 (27.68%)	105 (29.49%)	0.36	0.55	0.92 (0.68–1.23)
	C	418 (72.32%)	251 (70.51%)			
With family history	T	272 (30.56%)	23 (23.96%)	1.8	0.18	1.40 (0.86–2.28)
	C	618 (69.44%)	73 (76.04%)			
Without family history	T	272 (30.56%)	134 (30.45%)	0	0.97	1.01 (0.78–1.29)
	C	618 (69.44%)	306 (69.55%)			

HC, healthy control; SZ, participants with schizophrenia.

Finally, co-dominant, dominant, and recessive model analyses showed no significant differences in genotype or allele frequencies in the population of schizophrenia patients with a family history of schizophrenia compared to the healthy controls (P > 0.05). There were also no significant differences in the genotype and allele frequency distribution between patients with schizophrenia without a family history and the healthy controls (P > 0.05).

## Discussion

Although a patient’s genetic background appears to play an important role in the susceptibility to developing schizophrenia, the specific genes that confer increased risk are currently unknown. However, dysregulation of TLR molecules is known to exacerbate the immunoinflammatory response and genetic variations in genes associated with the TLR pathway may increase the risk of infections (Chen et al., 2021). Both immunoinflammatory responses and immune activation during neurodevelopment may contribute to schizophrenia pathogenesis. Furthermore, the SNP rs3775291 in *TLR3* has been shown to be associated with susceptibility to central nervous system diseases (Barkhash et al., 2013). Accordingly, Venkatasubramanian and Debnath (2013) have proposed that TLRs are associated with the development and progression of some cases of schizophrenia.

In the current study, a gene fragment of the rs3775291 locus was sequenced using first-generation sequencing and the differences in genotype and allele frequencies distribution between patients with schizophrenia and the control group were analysed employing co-dominant, dominant, and recessive models (Table 2). The results showed no significant differences between the patients with schizophrenia and the control group, and overall, rs3775291 was not a risk factor for schizophrenia; that is, rs3775291 was not associated with schizophrenia susceptibility. Aflouk et al. (2025) employed PCR-RFLP technology to genotype 260 controls and 260 schizophrenia patients of Caucasian population and also found no association between rs3775291 and schizophrenia, which is consistent with our findings. In our study, the effects of sex, age, and family history on genotype and allele frequencies distribution were further analysed. The results showed that sex and family history had no significant effect on either genotype or allele frequency distribution, whereas age did. In individuals younger than 40 years, results of both co-dominant and dominant model analyses showed that the frequencies of TC and CC genotypes of rs3775291 sites in patients with schizophrenia were significantly higher than those in the control group; therefore, TC and CC genotypes were associated with susceptibility to schizophrenia. There was no significant difference among people older than 40 years.

Ma et al. (2016) conducted a meta-analysis to verify the effect of rs3775291 on age-related macular degeneration (AMD) and found using homozygous and recessive models that this SNP is associated with combined AMD, geographic atrophy, and neovascular AMD, with the TT genotype exhibiting a protective effect (Ma et al., 2016). Mouse experiments have confirmed that the T allele of rs3775291 has a protective effect against the onset of geographic atrophy in advanced AMD, which may be a result of inhibiting retinal pigment epithelial cell death (Yang et al., 2008). This correlation was also confirmed in a case-control series (Yang et al., 2008). These findings suggest that rs3775291 may be age-related in the development of AMD. Our current study in patients with schizophrenia also found that rs3775291 was associated with age. In individuals younger than 40 years of age, the TT genotype had a protective effect against the development of schizophrenia. This suggests that the effect of rs3775291 on schizophrenia has an age-related component and that the TT genotype has a protective effect against schizophrenia in younger populations.

In addition, prenatal infection is a recognized risk factor for schizophrenia, as it can activate the TLR3 pathway in the mother. With age, continuous activation of TLR3 can generate reactive oxygen species and reactive nitrogen species, leading to neuronal damage and apoptosis (Venkatasubramanian and Debnath, 2013). This process is closely associated with disease progression, brain structural alterations, and persistent cognitive decline in schizophrenia (Talukdar et al., 2021). Thus, abnormal TLR3 signaling during development and dysregulation of TLR3 signaling during aging jointly shape the risk of schizophrenia across the entire lifespan. Therefore, from development to aging, the TLR3 pathway serves as a bidirectional bridge connecting immune changes to schizophrenia susceptibility: during critical developmental windows, TLR3 signaling acts as a trigger that disrupts neural circuits and lays the groundwork for susceptibility; in adulthood, it functions as an inflammation-driven engine that continuously deteriorates brain function through immune-aging-related mechanisms. We speculate that the age-dependent differences in the association between rs3775291 and schizophrenia observed in this study may arise from these immune changes at different life stages.

## Conclusion

Our findings provide new insights for the diagnosis and treatment of patients with schizophrenia. However, the patients enrolled in this study did not include their age at first onset, and whether rs3775291 affects the age at first onset of schizophrenia requires further analysis. In addition, the sample size in this study was relatively small, especially for patients with a family history of schizophrenia. Subsequent studies with larger sample sizes are needed to verify this finding.

## Data Availability

The raw data supporting the conclusions of this article will be made available by the authors, without undue reservation.
